# Influence of Cover Crop Termination on Ground Dwelling Arthropods in Organic Vegetable Systems

**DOI:** 10.3390/insects11070445

**Published:** 2020-07-15

**Authors:** Laura Depalo, Giovanni Burgio, Serena Magagnoli, Daniele Sommaggio, Francesco Montemurro, Stefano Canali, Antonio Masetti

**Affiliations:** 1 Department of Agricultural and Food Sciences, Alma Mater Studiorum-Università di Bologna, Viale G. Fanin, 42, 40127 Bologna, Italy; laura.depalo@unibo.it (L.D.); giovanni.burgio@unibo.it (G.B.); serena.magagnoli4@unibo.it (S.M.); dsommaggio@tiscali.it (D.S.); 2 Research Centre for Agriculture and Environment, Council for Agricultural Research and Economics, CREA, 70125 Bari, Italy; francesco.montemurro@crea.gov.it; 3 Council for Agricultural Research and Economics (CREA), Research Centre for Agriculture and Environment, Via della Navicella, 2-4, 00184 Roma (RM), Italy; stefano.canali@crea.gov.it

**Keywords:** Araneae, Carabidae, Staphylinidae, green manure, in-line tillage, no-tillage, pitfall traps, roller crimper

## Abstract

A key aspect in cover crop management is termination before the cash crop is planted. The aim of this study was to assess the effects of termination methods on ground-dwelling arthropods. The conventional mechanical termination method—i.e., green manuring by means of a disc harrow—was compared to flattening using a roller crimper. Two different crop systems were investigated for two growing seasons; cauliflower was grown in autumn after the termination of a mixture of cowpea, pearl millet, and radish, and tomato was cropped in spring and summer after the termination of a mixture of barley and vetch. Ground beetles (Coleoptera: Carabidae), rove beetles (Coleoptera: Staphylinidae), and spiders (Araneae) were sampled by means of standard pitfall traps throughout the growing season of both cash crops. The roller crimper increased the overall abundance of ground beetles in the first growing season of both cash crops, whereas in the second year, no significant effect could be detected. Rove beetles were more abundant in plots where the cover crops were terminated by the roller crimper. Finally, green manuring increased the abundance of spiders, especially on the first sampling date after cover crop termination. Albeit different taxa showed different responses, the termination of cover crops by a roller crimper generally increased the abundance of ground dwelling arthropods. Given that most of the sampled species were generalist predators, their increased abundance could possibly improve biological control.

## 1. Introduction

Cover crops are one of the habitat manipulation practices most often adopted to improve sustainability in agricultural systems [1]. The inclusion of cover crops in crop rotations to break the cash crop sequence, and/or instead of a long bare fallow periods, may positively influence multiple ecosystem services. A number of studies have documented the positive effects of cover crops on yield [2], weed control [3], nutrient availability [4], soil erosion [5], and arthropod dynamics [6,7].

The presence and management of cover crops can also affect the abundance and diversity of beneficial arthropods, which play an important role in biological control of pests and delivery of other ecosystem services [8,9,10]. Indeed, the abundance of beneficial arthropods may be limited by the low diversity in the sequences of traditional crop rotations [11] and by the disturbance of farming practices [8]. In this context, some cover crops can be also considered as an agroecological pest management practice to increase the complexity of cropping systems and to provide resources for beneficial arthropods; for this reason, they may also be defined as agroecological service crops (ASC) [12].

Timing and method of termination before the cash crop is planted are key aspects of cover crop management. Several methods are available to manage cover crops. Besides the use of herbicides, a method characterized by a negative environmental impact, mechanical techniques have been developed within the framework of sustainable agriculture. Currently, soil incorporation or green manuring is considered the best standard, especially in organic production systems. Green manure implies a shallow tillage (15–20 cm depth, generally performed by a rotary hoe or a disc harrow), yet, incorporating the cover crop below the soil surface [13]. Flattening is an alternative mechanical termination method. This is a conservation no-tillage technique based on the use of a roller crimper to crush cover crops, thus, obtaining a natural mulch, which can contribute to both weed control and reduce soil erosion, but could also possibly provide food and shelter for arthropods and small vertebrates [14,15]. However, the no-till roller crimper method is characterized by a number of constraints (i.e., cover crop regrowth, N immobilization, poor planting of the next crop) which may increase the cultivation risks and reduce the yield of the next crop [14,16].

An extremely reduced tillage method applicable in Mediterranean vegetable organic cropping systems relies on the concept of in-line tillage roller crimper (IL-RC). This technique is based on the use of machinery developed by a combination of a roller crimper, sharp vertical disks, and coulters (or chisels) assembled in the same frame and operating in sequence. This machinery allows the cover crops to be flattened whilst simultaneously obtaining a transplanting furrow (20–30 cm deep and a few centimeters wide). The mulch layer remains in place and covers the soil surface, thus, providing the required agronomic and ecological services [17].

The cover crop residues left on the soil, together with a reduced tillage system, can affect the assemblages of ground-dwelling arthropods by providing shelter, favorable microclimatic conditions, and food resources, and by lowering disturbance [18]. Species that thrive in closed habitats, with high amounts of plant residues, and that can exploit the decomposer community for prey, are likely to be favored [19]. Increased abundance and diversity of ground-dwelling arthropods could promote biological control of pest insects that is especially valuable for organic growers [20]. For example, Magagnoli et al. [21] demonstrated that the method of cover crop termination may influence the predation rate of artificial caterpillars by ground beetles (Coleoptera Carabidae).

The aim of this study was to assess the effects of termination methods on ground-dwelling arthropod assemblages. The conventional mechanical termination method (i.e., green manure, hereafter referred to as GM) was compared to flattening using an IL-RC. To accomplish the objective of this study, we investigated the responses of ground beetles, rove beetles (Staphylinidae), and spiders (Araneae) to cover crop termination methods. These taxa were selected for their standard use as bioindicators and because they include several biological control agents [22,23,24,25,26,27,28]. Given that IL-RC termination reduces disturbance, as well as increasing the above-ground structural complexity in comparison with GM, we hypothesize that this method would have overall beneficial effects on edaphic fauna.

## 2. Materials and Methods

### 2.1. Sampling Site and Experimental Design

This study was conducted from May 2015 to August 2017 at the research farm ‘Azienda Sperimentale Metaponto’ (40°23′00.0″ N 16°48′26.1″ E) of the Research Centre for Agriculture and Environment, Council for Agricultural Research and Economics (CREA-AA), Metaponto (MT), in southern Italy. At this experimental farm, the adaptation of horticultural systems to extreme climatic events is being tested, since these phenomena are increasing in the Mediterranean area. All crops were cultivated following organic farming rules, including pest and disease management practices that were carried out across all plots.

The site is characterized by an “accentuated thermo-Mediterranean” climate (UNESCO-FAO, 1963), with winter temperatures which can fall below 0 °C and summer temperatures which can rise above 40 °C. The mean annual precipitation is slightly higher than 500 mm and rainfall is unevenly distributed during the year, being concentrated mainly in the winter months. Soil is classified as a Typic Epiaquert, according to the Soil Taxonomy definition [29], the clay and silt contents being 60% and 36%, respectively, and the average soil bulk density is 1350 kg m^−3^.

Two different cropping systems were investigated: cover crops cultivated in spring-summer followed by a winter cash crop and cover crops in grown in the winter period followed by a spring-summer cash crop. In the cauliflower (*Brassica oleracea* L. var. *botrytis)* cropping system (winter), the cover crops consisted of four different mixtures of *Vigna unguiculata* (L.) Walp., *Pennisetum glaucum* (L.) R. Br. and *Raphanus sativus* L. (Supplementary Material Table S1), which were grown during the spring-summer of 2015 and 2016, and terminated before the transplanting of the cauliflower. The cover crops were hand-sown on 22 April 2015 and 5 May 2016 and terminated on 29 July 2015 and 28 July 2016, respectively; the cauliflower was manually transplanted on 3 August 2015 and 2 August 2016, respectively. The distance between rows was 0.8 m and the distance between plants of the same row was 0.6 m for an overall density of approximately 2 plants m^−^^2^. Organic fertilizers were applied to the soil at 150 kg N ha^−^^1^. Fertilizers were applied only once, approximately 15–20 days before transplanting the cauliflower. According to local farming practices, the crop was rain-fed and no irrigation was applied.

In the tomato (*Solanum lycopersicum* L. var. *Donald)* cropping system (summer), the cover crop consisted of two different combinations of *Hordeum vulgare* L. and *Vicia sativa* L. or *Vicia faba* Pers. (Supplementary Material Table S1), which were grown in the autumn of 2016 and 2017 and terminated before the transplanting the tomato crop. The cover crops were hand-sown on 4 November 2015 and 25 October 2016. Termination of the cover crop was carried out at flowering on 15 April 2016 and 29 April 2017, respectively. Tomato seedlings were transplanted by hand on 28 April 2016 and on 5 May 2017 in rows 1 m apart (plant spacing: 40 cm; plant density: 25,000 plants ha^−^^1^) The tomato plants were fertilized with organic fertilizers following the same procedure and amount described for cauliflower. All treatments were irrigated with the same volume of water, calculated to reach 100% of available soil water at 0–40 cm depth. Following this procedure, approximately 3000 and 3300 m^3^ ha^−^^1^ of water were used during 2016 and 2017, respectively.

Each experiment was arranged in a randomized complete block design. In each growing season, four blocks were set up for cauliflower and six for tomato. Each block consisted of two plots where the same cover crop mixtures were grown and terminated by IL-RC or GM. The plots for the cauliflower and tomato respectively measured 6 × 4 m and 4 × 18 m and were separated from each other by a 2 m-wide strip of bare soil.

The IL-RC system incorporated features of rolling/crimping with in-line tillage by positioning coulters mounted on the front of the 2 m-wide roller crimper and chisels in-line on the rear. The coulters and chisels cut a strip of 3 to 5 cm through the mulch to provide adequate space for transplant placement. This flatted the cover crops and created a 20 to 30 cm deep transplanting furrow, leaving a mulch layer in place. At the time of IL-RC termination, the cover crops in the GM treatment were chopped and ploughed to a 15 to 20 cm depth to prepare for transplanting [30].

### 2.2. Arthropod Sampling

To investigate soil arthropods, pitfall traps were set following standard practices available in the literature [23,31,32]. Each trap consisted of two collecting cups (600 mL; 10 cm diameter) connected by a 10 cm-high and 1 m-long Plexiglas barrier. The two cups of each trap were buried at the sides of a cash crop row so that the barrier crossed the row. Plastic lids were placed above the cups both to reduce bycatches and avoid overflow in case of heavy rainfall. Cups were filled with 40% aqueous solution of propylene glycol as a killing and preservative agent.

Pitfall traps were set approximately four weeks after the cash crop had been transplanted. In cauliflower, two traps per plot were maintained continuously for the whole growing season, i.e., from mid-September to the end of November in both 2015 and 2016. Traps were serviced every three weeks and four collections of arthropods were carried out over an 84-day period. In tomato, one trap per plot was maintained for 7 out of 21 days from mid-May to late July in 2016 and 2017. The 7 day trapping period was planned to avoid dryness in the traps due to high temperatures in the summer months. Four collections of arthropods were also carried out in tomato.

The arthropods collected were stored at 4 °C prior to identification in the laboratory. Ground beetles (Coleoptera: Carabidae), rove beetles (Coleoptera: Staphylinidae), and spiders (Araneae) were counted for each sample. All beetles were identified to species level, except for the individuals belonging to the subfamily Aleocharinae (Staphylinidae), since adequate taxonomic literature is not available [33]. Spiders were sorted to family level.

### 2.3. Statistical Analysis

The two growing seasons were considered separately for each cover crop–cash crop system in all the analyses.

A generalized linear mixed model (GLMM) analysis was carried out using the R Language, version 3.5.0, package “lme4”. In this analysis, the number of individuals of each macrogroup (i.e., Carabidae, Staphylinidae, and Araneae) was considered as dependent variable; termination method (IL-RC and GM) was used as a fixed factor, while sampling dates were considered as repeated measures and blocks as a random factor. Different data structures were considered for the different taxa, and final selection of an error distribution was based on the lowest values of Akaike’s information criteria. The data were fitted by maximum likelihood (Laplace Approximation) to a negative binomial generalized linear mixed model (GLMM) with a log link function. No correction for overdispersion was used after a dispersion check of data had been performed with “blmeco” package R Language, version 3.5.0. In the case of a significant interaction “termination method*sampling date”, a GLMM with the same features as previously described was performed separately for each sampling date.

The dominant species/genera of Carabidae and Staphylinidae (which pooled, accounted for approximately 60% of the total number of catches of their group) were analyzed by a two-way ANOVA. In this analysis, the dependent variable was the total abundance in each plot in the whole growing season, while the sources of variation were termination method and block (R Language, version 3.5.0, package “car”). Data not matching the assumption of normality were log transformed before analysis. When the block effect was not significant, it was removed from the model and the data were analyzed as a complete randomized design.

## 3. Results

### 3.1. Macrogroups in Cauliflower

**Figure 1:**
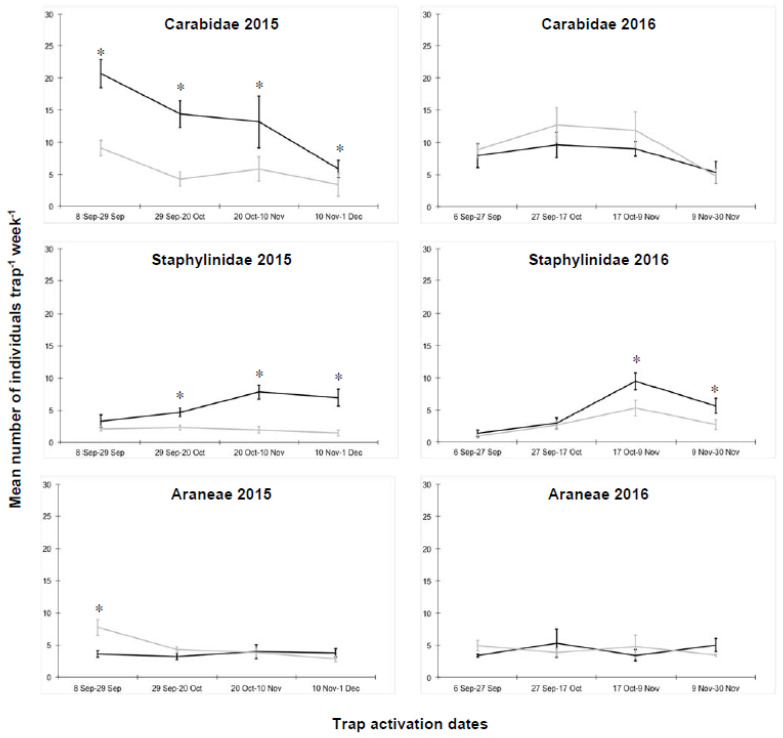
Abundance of individual Carabidae, Staphylinidae and Araneae collected in the experimental plots of cauliflower, where the cover crops were terminated either with a roller crimper (IL-RC, black line) or through green manuring (GM, grey line). Vertical bars represent standard errors of the means; * = significant differences within each sampling date as detected by the generalized linear mixed model.

In the cauliflower plots, 1836 and 1653 ground beetles, 734 and 743 rove beetles, and 692 and 757 spiders were collected in 2015 and 2016, respectively (Supplementary Material Tables S2–S4). In 2015, the termination technique significantly influenced the arthropod abundances (Table 1). A higher abundance of ground beetles was detected in IL-RC plots, during the whole season (Figure 1). The abundance of rove beetles increased over time in IL-RC, but not in GM, thus, explaining the significant interaction between termination technique and sampling date (Table 1). Significant differences between IL-RC and GM plots were detected from the second sampling date until the end of the experiment. In addition, for spiders, a significant interaction between termination technique and sampling date was observed; the mean number of individuals per plot was higher in GM than IL-RC only at the first sampling date (Figure 1).

In 2016, no significant difference between the termination techniques could be detected for ground beetles and spiders (Table 1). Rove beetles showed a pattern similar to the previous year, with significantly higher abundances in IL-RC than in GM on the third and fourth sampling dates (Figure 1).

### 3.2. Dominant Species in Cauliflower Plots

**Figure 2:**
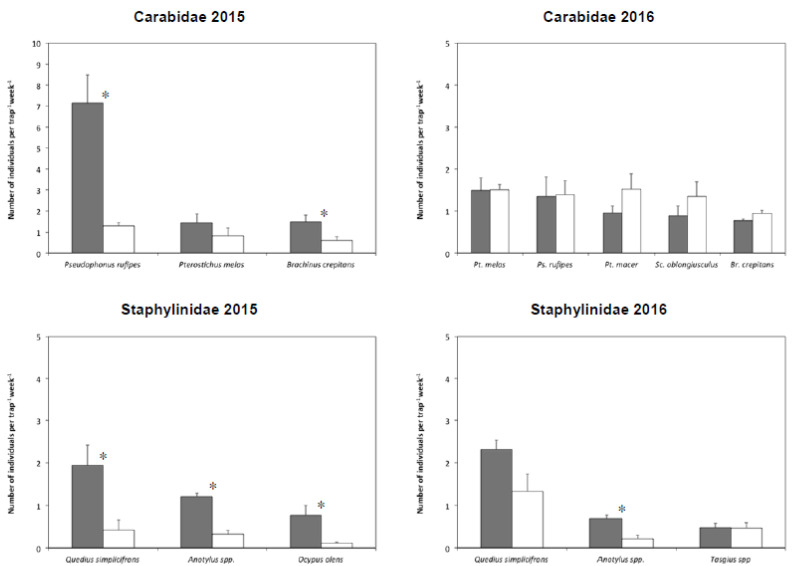
Mean number of individuals caught per trap per week of the dominant species of Carabidae and Staphylinidae collected in the experimental plots of cauliflower, where the cover crops were terminated either with a roller crimper (IL-RC, dark grey bars) or through green manuring (GM, white bars). Lines represent the standard errors of the means; * = significant differences for each species as detected by ANOVA.

In the cauliflower plots, 33 species of ground beetle were collected in 2015 and 32 in 2016 (Supplementary Material Table S2). In 2015, the three most abundant species, which accounted for 67% of the individuals, were considered for data analysis. The dominant species (47% of the total), *Pseudophonus rufipes* (De Geer) and *Brachinus crepitans* (L.), were positively influenced by IL-RC (Figure 2; Table 2), while for *Pterostichus melas* (Creutzer), no differences were detected between termination methods.

In 2016, five species (*P. melas*, *P. rufipes*, *Pterostichus macer* (Marsham), *Scybalicus oblongiusculus* (Dejean), and *B. crepitans*) accounted for nearly 70% of the total individuals collected. Only for *B. crepitans* was there a nearly significant difference in favor of GM (P = 0.07), while for the other species, there was no significant difference between the termination techniques (Figure 2; Table 2).

In 2015 and 2016, 36 and 26 species/genera of rove beetle were identified, respectively (Supplementary Material Table S3). In the first year, the three most abundant species *Quedius simplicifrons* Fairmaire, *Anotylus* spp. and *Ocypus olens* (O. Müller), together accounting for 63% of the total catches, were all positively influenced by IL-RC termination (Figure 2; Table 3). In the second year, three species/genera accounted for approximately 70% of the total individuals collected. *Anotylus* spp. was significantly more abundant in IL-RC plots in comparison with GM ones. Although the level of statistical significance was 0.07, the abundance of *Q. simplicifrons* in IL-RC plots was almost twice that in GM plots. Finally, abundance of the genus *Tasgius* spp. did not show any difference between the two termination techniques (Figure 2; Table 3).

### 3.3. Macrogroups in Tomato Plots

**Figure 3:**
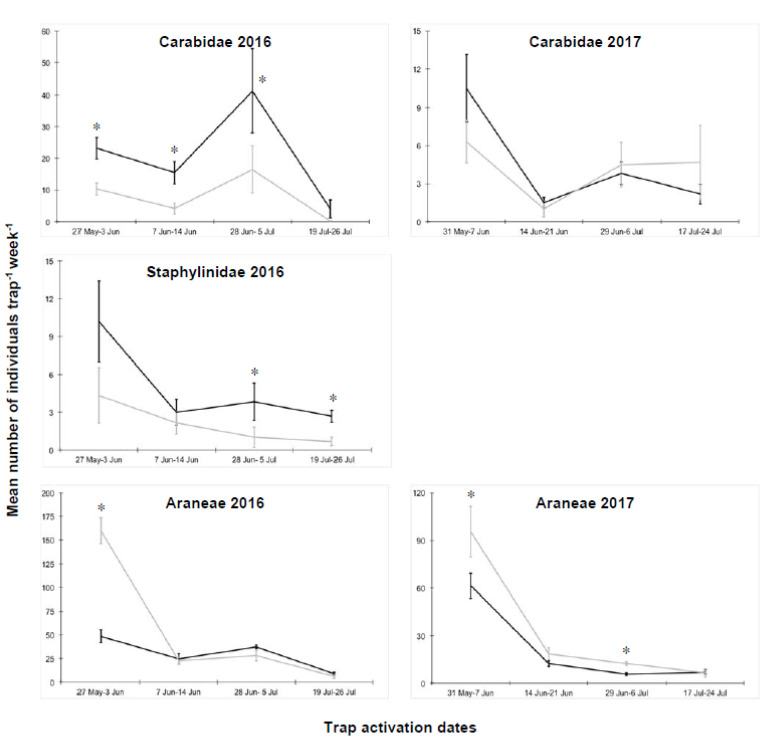
Abundance of individual Carabidae, Staphylinidae, and Araneae collected in the experimental plots of tomato, where the cover crops were terminated either with a roller crimper (IL-RC, black line) or through green manuring (GM, grey line). Vertical bars represent standard errors of the means; * = significant differences within each sampling date as detected by the generalized linear mixed model. Note that ordinate scales differ among the charts.

In the tomato plots, 684 and 207 ground beetles, 167 and 31 rove beetles, and 1795 and 1224 spiders were collected in 2016 and 2017, respectively (Supplementary Material Tables S2–S4). In the first year of the experiment, significant differences between termination techniques were detected for all taxa (Table 4). The adoption of IL-RC termination positively influenced the abundance of both ground and rove beetles, while spiders were more abundant in GM plots, but only at the first sampling date (Figure 3). As with the experiment on cauliflower, ground beetles were generally more abundant in IL-RC plots, with significant differences on the first three sampling dates. Rove beetles were significantly more abundant in IL-RC plots on the last two sampling dates (Figure 3).

In the summer of 2017, beetle abundance was very low, in comparison with the previous year, which is likely to be due to the severely hot and dry conditions (from mid-April to end July 2016, 122.9 mm of total rainfall and a mean maximum temperature of 27.3 °C were recorded, whereas for the same period in 2017, 57.1 mm of total rainfall and 28.2 °C were recorded). In particular, very few rove beetles were trapped and a robust statistical analysis could not be performed on this taxon. Ground beetle abundance did not show any significant difference between termination techniques; whereas a higher abundance of spiders was found in GM plots in comparison with IL-RC plots, on the first and third sampling dates (Figure 3; Table 4).

### 3.4. Dominant Species in Tomato Plots

**Figure 4:**
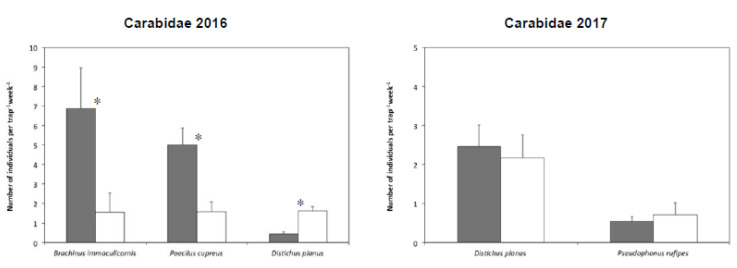
Mean number of individuals caught per trap per week of the dominant species of Carabidae collected in the experimental plots of tomato, where the cover crops were terminated either with a roller crimper (IL-RC, dark grey bars) or through green manuring (GM, white bars). Lines represent the standard errors of the means; * = significant differences for each species as detected by ANOVA.

Overall, 15 species of ground beetle were collected in the tomato plots both in 2016 and 2017 (Supplementary Material Table S2). In 2016, data on the three most abundant species, accounting for approximately 0% of the individuals collected, were analyzed. The abundance of all the species was significantly influenced by the termination method; *Brachinus immaculicornis* Dejean and *Poecilus cupreus* (L.) were more abundant in the IL-RC plots, whereas *Distichus planus* (Bonelli) was favored by GM (Figure 4; Table 5). In 2017, the two dominant species, *D. planus* and *P. rufipes*, represented more than 70% of the individuals collected, but both of them were equally abundant in plots subjected to the two termination techniques. For the rove beetles, 23 and 8 species/genera were identified in 2016 and 2017, respectively (Supplementary Material Table S3). Due to the small numbers of individuals, a species analysis was not carried out on this taxon.

## 4. Discussion

Overall, the cover crop termination techniques of cover crops strongly affected the abundances of the soil arthropods considered in this study, with specific responses influenced by taxa and sampling dates. In particular, the in-line tillage termination method (IL-RC) showed a generally positive influence on the abundance of ground beetles and rove beetles in comparison with GM, both at family level and for the numerically dominant species.

The abundance of ground beetles showed an inconsistent response across the two growing seasons of the same cash crop. In the first year, their abundance was generally favored by flattened cover crops in both the cauliflower and tomato plots, whereas no differences were detected between termination techniques in the second year. The beneficial effect of reduced/no-tillage conditions on the abundance of ground beetles is well known and our results corroborate the findings of House and Parmelee [34] and Brévault et al. [35], in sorghum and cotton, respectively. Similar results were obtained by Tamburini et al. [36] in winter cereal crops, where conservation tillage supported a greater abundance of ground-dwelling predators, including ground beetles, in comparison with conventional tillage.

The analysis at species level showed that the total abundance of *P. rufipes*, the dominant species in the cauliflower experiment, strikingly changed across years and was positively influenced by use of IL-RC only in the first year. This omnivorous beetle is often recorded as the dominant species in studies carried out in Mediterranean agroecosystems [28,31,37]. Several studies investigated the response of *P. rufipes* to tillage, and both positive and negative effects were reported [27,28,38,39,40].

Adults of *Brachinus* spp. are considered to be predators, whereas larvae are parasitoids, with some species exploiting beetle pupae including other ground beetles such as *Amara* [41]. In our study, *B. crepitans* in the cauliflower plots and *B. immaculicornis* in the tomato plots were favored by the IL-RC in the first growing season. To our knowledge, no previous studies are available on the response of this genus to cover crop management and tillage operations.

*Poecilus cupreus* in the tomato plots was favored by the no-tillage IL-RC. Our findings confirm the results of other studies, indicating a negative effect of tillage on this species [27,39].

*Distichus planus* was more abundant in GM during the first year of the experimental trial on tomato. This is a predator common in sandy soil near to the seaside [42]. In total, 161 out of 173 (93.1%) individuals were caught in the tomato plots and the higher abundance in GM could be linked to the preference of this beetle for loose soil produced by tillage operations.

Among the taxa studied, rove beetles responded more consistently to the different termination techniques. During both years of the cauliflower experiment and in the first year of the tomato experiment, this family clearly showed a higher abundance in IL-RC plots. This is in line with a study by Jackson and Harrison [6], which reported an increased activity density of rove beetles in plots where the cover crops were killed by an herbicide vs. conventional tillage. It has been demonstrated that ecological infrastructures (e.g., hedgerows and cover crops) within cropping systems can enhance the abundance and diversity of rove beetles by providing prey and refuge sites [43]. The density of rove beetles was observed to increase when mown grass was left to dry in experimental plots, suggesting that plant residues could be important for their persistence [44].

The abundance of the dominant rove beetle species in the cauliflower plots showed a similar pattern during both growing seasons, responding positively to IL-RC, except for the genus *Tasgius*. *Quedius simplicifrons* and *O. olens* are large predators (>12 mm) and the greater abundance of these species following IL-RC termination could be attributed to a general higher sensitivity of larger organisms to tillage operations, which has been stressed in a review by Kladivko [45]. The genus *Anotylus* includes mostly saprophagous species [46] and its increased presence in IL-RC plots could be linked to the wider availability of decaying organic matter left on the soil surface after flattening a cover crop.

The ecological specialization and the sensitivity to environmental changes make rove beetles a suitable and useful bioindicator in agroecosystems. Although rove beetles have been considered in some cases to be more sensitive than ground beetles for the assessment of anthropogenic disturbances [47,48], their utility in biomonitoring during this study was limited due to difficulties of species identification.

The effect of cover crop termination technique on spiders was generally more pronounced on the first sampling dates, when GM favored this group in the first year of the cauliflower experiment and in both years of the tomato experiment. Other studies showed increased spider abundance in no-tillage [49,50] or conservation tillage [36] plots compared to conventional tillage. The green manuring (GM) in our experiment can be included amongst conservation methods, since it is characterized by reduced tillage and is adopted in organic farming.

As is typical for pitfall traps, in our experiment, catches were dominated by wolf spiders (Lycosidae) (Supplementary Material Table S4). The lower abundance of wolf spiders in IL-RC compared with GM plots could be due to the mulch layer interfering with movement and pursuit of prey by this taxon. The plant residues left by the IL-RC treatment decay over time and this could explain the trend for spiders, where a large difference between treatments on the first sampling date tends to level off. Moreover, the inclusion of cover crop residues in the soil, as provided by GM, could have enhanced the detrital food chain, which has been shown to be relevant for spiders [51].

Although there was a wide range of responses between groups and species, our study indicated an overall tendency for a greater abundance of soil dwelling arthropods following application of the IL-RC method. Very few studies are available on the effect of roller crimper termination on arthropods in vegetable crops, but our results are in agreement with the findings by Jackson and Harrison [6] who worked on sweet potato crops. Our results are also in line with those of Magagnoli et al. [21], who detected an increase in predation by soil-living beetles in plots where vetch had been flattened by a rolled crimper, whereas there were no differences between termination methods when barley was used as cover crop. This suggests that the effects of different termination methods can also be influenced by the cover crop species/mixtures used in the rotation, as confirmed by Rivers [20] and Pisani Gareau [52]. The differential effects of cover crop species on ground dwelling arthropods fall outside the aims of this study. However, we assume that the impact of different cover crop mixtures is negligible in our experiments. Firstly, pitfall traps were deployed four weeks after cover crop termination. Moreover, the dispersal abilities of most taxa allowed them to move easily between small plots, overshadowing the influence of different mixtures of cover crops.

The underlying ecological mechanisms leading to habitat responses by the taxa sampled in this study are largely unknown. Overall, the indirect effects of the termination practices may have had a stronger impact on arthropod population dynamics than direct mortality or emigration due to tillage. Given that pitfall traps were deployed four weeks after the cover crops were killed, the direct mortality and emigration due to cover crop termination does not seem to be a major factor explaining the different responses of taxa. The relationships between most of the arthropod sampled and habitat features are poorly understood, because multiple and potentially competing mechanisms may combine to drive abundance and diversity patterns [53,54,55]. These mechanisms include prey density, location and capture, refugia from predation and cannibalism, microclimatic conditions, and access to plant resources to supplement carnivorous diet (e.g., weed seeds, pollen or nectar).

## 5. Conclusions

Our results contribute to a more complete assessment of the effects of cover crop termination, which usually relies on agronomic evaluations. Considering that the IL-RC technology was developed mainly to control weeds in organic systems, it is important to stress that this termination method can improve the overall abundance of soil arthropods, which are considered important providers of ecosystem services. Given that most of the species sampled are predators, they could contribute mainly to biological control. From the stand point of organic farming, it is important to consider carefully whether the approach to tillage may have important implications for the ecological services provided by ground-dwelling arthropods; more research is needed to better understand the dynamics of the different groups.

## Figures and Tables

**Table 1:** Statistics of the model effect for the negative binomial General Linear Mixed Model carried out on the number of individuals Carabidae, Staphylinidae, and Araneae in cauliflower plots.

Cauliflower	Carabidae			Staphylinidae			Spiders		
2015	df	*Χ* ^2^	*p*	df	*Χ* ^2^	*p*	df	*Χ* ^2^	*p*
Treatment	1	52.12	<0.001	1	58.97	<0.001	1	5.00	<0.05
Sampling date	3	46.18	<0.001	3	6.46	0.09	3	17.41	<0.001
Treatment × Sampling date	3	3.86	0.28	3	11.93	<0.01	3	16.89	<0.001
**2016**	**df**	***Χ*^2^**	***p***	**df**	***Χ*^2^**	***p***	**df**	***Χ*^2^**	***p***
Treatment	1	1.88	0.16	1	10.69	<0.01	1	0.0001	0.99
Sampling date	3	28.16	<0.001	3	72.76	<0.001	3	0.33	0.95
Treatment × Sampling date	3	1.57	0.67	3	3.52	0.32	3	5.45	0.14

**Table 2:** Results of ANOVA carried out on the most abundant species of Carabidae (pooling all the individuals caught in each plot over the whole sampling period) in cauliflower plots. Blocks were omitted if their effect was not significant.

Cauliflower	*Pseudophonus rufipes*			*Pterostichus melas*			*Brachinus crepitans*			*Scybalicus oblongiusculus*			*Pterostichus macer*		
2015	df	F	*p*	df	F	*p*	df	F	*p*	*-*			-		
Treatment	1	57.08	<0.001	1	9.52	0.053	1	6.70	<0.05	-			-		
Block				3	13.55	<0.05				-			-		
**2016**	**df**	**F**	***p***	**df**	**F**	***p***	**df**	**F**	***p***	**df**	**F**	***p***	**df**	**F**	***p***
Treatment	1	0.003	0.96	1	0.08	0.79	1	4.95	0.07	1	1.26	0.30	1	1.94	0.21

**Table 3:** Results of ANOVA carried out on the most abundant species of Staphylinidae (pooling all the individuals caught in each plot over the whole sampling period) in cauliflower plots.

Cauliflower	*Quedius simplicifrons*			*Anotylus* spp.			*Ocypus olens*		
2015	df	F	*p*	df	F	*p*	df	F	*p*
Treatment	1	8.14	<0.05	1	63.25	<0.001	1	9.10	<0.05
**Cauliflower**	***Quedius simplicifrons***			***Anotylus* spp.**			***Tasgius* spp.**		
**2016**	**df**	**F**	***p***	**df**	**F**	***p***	**df**	**F**	***p***
Treatment	1	4.81	0.07	1	19.96	<0.01	1	0.004	0.95

**Table 4:** Statistics of the model effect for the negative binomial General Linear Mixed Model carried out on the number of individuals Carabidae, Staphylinidae, and Araneae in tomato plots. Staphylinidae in 2017 were not analyzed because of the limited number of caught individuals.

Tomato	Carabidae			Staphylinidae			Spiders		
2016	df	*Χ* ^2^	*p*	df	*Χ* ^2^	*p*	df	*Χ* ^2^	*p*
Treatment	1	21.76	<0.001	1	10.88	<0.001	1	5.39	<0.05
Sampling date	3	46.77	<0.001	3	17.34	<0.001	3	266.65	<0.001
Treatment × Sampling date	3	4.25	0.24	3	2.21	0.53	3	45.40	<0.001
**2017**	**df**	***Χ*^2^**	***p***	**df**	***Χ*^2^**	***p***	**df**	***Χ*^2^**	***p***
Treatment	1	0.011	0.92	-	-	-	1	10.94	<0.001
Sampling date	3	21.64	<0.001	-	-	-	3	300.36	<0.001
Treatment × Sampling date	3	3.47	0.32	-	-	-	3	4.84	0.18

**Table 5:** Results of ANOVA carried out on the most abundant species of Carabidae (pooling all the individuals caught in each plot over the whole sampling period) in cauliflower plots.

Tomato	*Distichus planus*			*Poecilus cupreus*			*Brachinus immaculicornis*		
2016	df	F	*p*	df	F	*p*	df	F	*p*
Treatment	1	26.85	<0.001	1	11.40	<0.01	1	5.287	<0.05
**Tomato**	***Distichus planus***			***Pseudophonus rufipes***			**-**		
**2017**	**df**	**F**	***p***	**df**	**F**	***p***	**-**		
Treatment	1	0.13	0.73	1	0.25	0.63	-		

## Supplementary Materials

The following are available online at https://www.mdpi.com/2075-4450/11/7/445/s1, Table S1: Compositions and seeding rates of the cover crops, Table S2: List of ground beetle species, Table S3: List of rove beetle species, Table S4: List of spider families.
