# Supplementary material for: Influence of Cover Crop Termination on Ground Dwelling Arthropods in Organic Vegetable Systems
Source: Insects. 2020 Jul 15;11(7):445. doi: 10.3390/insects11070445 (PMC7412336; doi:10.3390/insects11070445)
Supplement: Supplementary file 1 [file insects-11-00445-s001.pdf]

**Supplementary material Table S1.** Compositions and seeding rates of the cover crops.

| **Cauliflower** |
|---|
| 100% *Vigna unguiculata*<br>70% *Vigna unguiculata* + 30% *Pennisetum glaucum*<br>50% *Vigna unguiculate* + 50% *Pennisetum glaucum*<br>40% *Vigna unguiculata* + 30% *Pennisetum glaucum* + 30% *Raphanus sativus* |
| **Seeding rates**<br>*Vigna unguiculata* 50 kg $ha^{-1}$<br>*Pennisetum glaucum* 60 kg $ha^{-1}$<br>*Raphanus sativus* 50 kg $ha^{-1}$ |
| **Tomato** |
| 20% *Hordeum vulgare* + 80% *Vicia sativa*<br>20% *Hordeum vulgare* + 80% *Vicia faba* Pers. |
| **Seeding rates**<br>*Hordeum vulgare* 40 kg $ha^{-1}$<br>*Vicia sativa* 65 kg $ha^{-1}$<br>*Vicia faba* 120 kg $ha^{-1}$ |

**Supplementary material Table S2.** List of ground beetle species sampled by pitfall traps. GM = green manure, RC = roller crimper.

| | Cauliflower | | | | | Tomato | | | | |
|---|---|---|---|---|---|---|---|---|---|---|
| | 2015 | | 2016 | | | 2016 | | 2017 | | |
| **Species** | **RC** | **GM** | **RC** | **GM** | **Total** | **RC** | **GM** | **RC** | **GM** | **Total** |
| *Acinopus megacephalus* (Rossi) | | | | | | 1 | | | | 1 |
| *Agonum* sp. | | | 3 | 1 | 4 | | | | | |
| *Amblystomus* sp. | | | 1 | 2 | 3 | | | | | |
| *Anchomenus dorsalis* (Pontoppidan) | 18 | 16 | 29 | 29 | 92 | 37 | 7 | | | 44 |
| *Philochthus lunulatus* (Fourcroy) | | | | | | 1 | | | | 1 |
| *Brachinus crepitans* (L.) | 144 | 59 | 66 | 91 | 360 | 34 | 3 | | 1 | 38 |
| *Brachinus elegans* Chaudoir | | 1 | | | 1 | | | | | 0 |
| *Brachinus immaculicornis* Dejean | 16 | 10 | 24 | 29 | 79 | 165 | 37 | | 3 | 205 |
| *Brachinus italicus* Dejean | 1 | 2 | 9 | 10 | 22 | 2 | | | | 2 |
| *Brachinus psophia* Audinet-Serville | | | | 3 | 3 | 2 | | 1 | | 3 |
| *Calathus cinctus* Motschulsky | 86 | 62 | 31 | 71 | 250 | 1 | | | | 1 |
| *Calathus circumseptus* Germar | 9 | 2 | 3 | 2 | 16 | 2 | | | | 2 |
| *Calathus fuscipes* (Goeze) | 1 | | 3 | 2 | 6 | | | | | |
| *Campalita maderae* (Fabricius) | 1 | | | | 1 | | | | | |
| *Carabus coriaceus* L. | 11 | 6 | 35 | 24 | 76 | | | | | |
| *Carterus fulvipes* (Latreille) | | | | | | | | 2 | | 2 |
| *Chlaenius chrysocephalus* (Rossi) | 2 | | | | 2 | | | | | |
| *Chlaenius festivus* (Panzer) | | | | | | 4 | | | | 4 |
| *Demetrias atricapillus* (L.) | | | | | | 5 | | | | 5 |
| *Dicheirotrichus chloroticus* (Dejean) | 11 | 21 | 34 | 64 | 130 | 0 | | | | |
| *Dinodes decipiens* (Dufour) | 1 | | 4 | 4 | 9 | 15 | 1 | 6 | 12 | 34 |
| *Distichus planus* (Bonelli) | 4 | 1 | 5 | 2 | 12 | 11 | 39 | 59 | 52 | 161 |
| *Harpalophonus italus* (Schaum) | | | 1 | | 1 | | | | | |
| *Harpalus distinguendus* (Duftschmid) | 24 | 8 | 2 | 5 | 39 | | | | | 1 |
| *Harpalus oblitus* Dejean | | | | | | | | | | |
| *Laemostenus acutangulus* (Schaufuss) | 1 | | | | 1 | | | | | |
| *Lebia humeralis* Dejean | 4 | 4 | | 1 | 9 | 1 | | 3 | 4 | 8 |
| *Leistus fulvibarbis* Dejean | 2 | 2 | 1 | | 5 | | | | | |
| *Licinus silphoides* (Rossi) | | | 7 | 3 | 10 | | | | | |
| *Microlestes corticalis* (Dufour) | 3 | 7 | | | 10 | 2 | | | | 2 |
| *Microlestes fulvibasis* (Reitter) | | | | | | | | 5 | 3 | 8 |
| *Microlestes* cfr. *fissuralis* (Reitter) | | | | | | 1 | | | | 1 |
| *Nebria brevicollis* Motschulsky | 2 | 2 | | 3 | 7 | | | | | |
| *Notiophilus geminatus* Dejean | 4 | | | | 4 | | | | | |
| *Paratachys micros* (Fischer) | | 1 | | | 1 | | | | | |
| *Parophonus hispanus* (Rambur) | | | | | | | 1 | | | 1 |
| *Pedius inquinatus* (Sturm) | | | 4 | 1 | 5 | 2 | 1 | 1 | | 4 |
| *Poecilus cupreus* (Linnaeus) | | | 20 | 16 | 36 | 120 | 38 | | | 158 |
| *Poecilus cursorius* (Dejean) | | 1 | | 1 | 2 | 24 | 20 | | 1 | 45 |
| *Poecilus nitidus* (Dejean) | 9 | 18 | 1 | | 28 | 18 | 16 | | | 34 |
| *Poecilus versicolor* (Sturm) | 12 | 10 | | | 22 | | | | | |
| *Polyderis algiricus* (Lucas) | 1 | | | | 1 | | | | | |
| *Pseudophonus griseus* (Panzer) | 24 | 6 | 4 | 1 | 35 | | | | | |
| *Pseudophonus rufipes* (Degeer) | 685 | 123 | 124 | 133 | 1065 | 27 | 8 | 13 | 17 | 65 |
| *Pterostichus macer* (Marsham) | 27 | 25 | 90 | 146 | 288 | 3 | 2 | | | 5 |

**Supplementary material Table S2.** List of ground beetle species sampled by pitfall traps. GM = green manure, RC = roller crimper. (continued)

| | Cauliflower | | | | | Tomato | | | | |
|---|---|---|---|---|---|---|---|---|---|---|
| | 2015 | | 2016 | | | 2016 | | 2017 | | |
| **Species** | **RC** | **GM** | **RC** | **GM** | **Total** | **RC** | **GM** | **RC** | **GM** | **Total** |
| *Pterostichus melas* (Creutzer) | 139 | 81 | 137 | 145 | 502 | 7 | 2 | 1 | 1 | 11 |
| *Scybalicus oblongiusculus* (Dejean) | 48 | 60 | 85 | 130 | 323 | 4 | 6 | 15 | 4 | 29 |
| *Siagona europaea* Dejean | 1 | 4 | 4 | 1 | 10 | 1 | 3 | 1 | 1 | 6 |
| *Stenolophus teutonus* (Schrank) | | | | | | 1 | 2 | | | 3 |
| *Syntomus obscuroguttatus* (Duftschmid) | | | | | | 2 | | | | 2 |
| *Trechus quadristriatus* (Schrank) | 12 | 1 | 2 | 3 | 18 | 4 | 1 | | | 5 |
| *Zabrus tenebrioides* (Goeze) | | | | 1 | 1 | | | | | |
| **Total** | **1303** | **533** | **729** | **924** | **3489** | **497** | **187** | **108** | **99** | **891** |

**Supplementary material Table S3.** List of rove beetle species sampled by pitfall traps. GM = green manure, RC = roller crimper.

| | Cauliflower | | | | | Tomato | | | | |
|---|---|---|---|---|---|---|---|---|---|---|
| | 2015 | | 2016 | | | 2016 | | 2017 | | |
| **Species** | **RC** | **GM** | **RC** | **GM** | **Total** | **RC** | **GM** | **RC** | **GM** | **Total** |
| Aleocharinae | 51 | 78 | 43 | 40 | 212 | 12 | 50 | 5 | 14 | 81 |
| *Anotylus* sp.1 | 29 | 112 | 21 | 66 | 228 | | | | | |
| *Anotylus* sp.2 | 2 | 6 | | | 8 | | | 2 | 1 | 3 |
| *Anotylus* sp.3 | | | | 1 | 1 | | | | | |
| *Astenus bimaculatus* (Erichson) | 2 | 1 | 2 | 1 | 6 | 1 | 2 | | | 3 |
| *Astenus lyonessius* (Joy) | 1 | 3 | | 1 | 5 | 2 | 2 | | | 4 |
| *Astrapaeus ulmi* (Rossi) | | 1 | | | 1 | 1 | 2 | | | 3 |
| *Bisnius sordidus* (Gravenhorst) | | 1 | | | 1 | | | | | |
| *Bisnius* sp.1 | | | | | | | | | 1 | 1 |
| *Bisnius/Rabigus* sp.1? | 3 | 4 | | | 7 | | | | | |
| *Carpelimus corticinus* (Gravenhorst,) | 12 | 8 | 6 | 2 | 28 | 3 | 9 | | | 12 |
| *Cordalia obscura* (Gravenhorst) | | | | | | | | | 1 | 1 |
| damaged specimens | 1 | | 3 | 5 | 9 | | | | | |
| *Domene* sp. | | 1 | | | 1 | | | | | |
| *Gabrius* sp. | | | | | | | | | | 1 |
| *Ischnosoma splendidum* (Gravenhorst,) | | | 2 | | 2 | | | | | |
| *Leptacinus intermedius* Donisthorpe | 2 | 2 | | 1 | 5 | 1 | 2 | | 1 | 4 |
| *Leptobium gracile* (Gravenhorst) | 1 | | | 2 | 3 | | 2 | | | 2 |
| *Lobrathium* sp. | | | | | | | | 1 | | 1 |
| *Luzea nigritula* (Erichson) | 2 | 5 | | 2 | 9 | | 1 | | | 1 |
| *Megalinus glabratus* (Gravenhorst) | | 1 | | 1 | 2 | 1 | | | | 1 |
| *Mycetoporus* sp. | | | 8 | 4 | 12 | 2 | 2 | | | 4 |
| *Ocypus nitens* (Schrank) | 1 | | 9 | 11 | 21 | | 1 | | | 1 |
| *Ocypus olens* (O. Muller) | 10 | 75 | 6 | 38 | 129 | | | | | |
| *Othius volans* J. Sahlberg | | 1 | | | 1 | | | | | |
| *Philonthus cognatus* Stephens | | | | | | 2 | 1 | | | 3 |
| *Philonthus concinnus* (Gravenhorst) | | 3 | | 2 | 5 | 8 | 5 | | | 13 |
| *Platystethus nitens* (C. Sahlberg) | 1 | 8 | 1 | 2 | 12 | 3 | 5 | | | 8 |
| *Platystethus* sp. | 0 | 2 | | | 2 | | | | | |
| Pselaphinae sp.1 | | | | | | | 2 | | | 2 |
| Pselaphinae sp.2 | 1 | | | | 1 | | | | | |
| Pselaphinae sp.3 | 1 | | | | 1 | | | | | |
| *Pseudolathra lusitanica* (Erichson) | | | | | | 1 | | | | 1 |
| *Quedius levicollis* (Brullé) | | 4 | | | 4 | | | | | |
| *Quedius picipes* (Mannerheim) | | 1 | 1 | 1 | 3 | | | | | |
| *Quedius simplicifrons* Fairmaire | 41 | 186 | 129 | 222 | 578 | | | | | |
| *Rugilus orbiculatus* (Paykull) | 1 | 1 | | 7 | 9 | 3 | 11 | | | 14 |
| *Scopaeus debilis* Hochhuth | 4 | 1 | | | 5 | | | | | |
| Scydmaenidae | | | | | | | | 1 | 1 | 2 |
| Staphylinidae sp. | | | | | | 2 | | | | 2 |
| *Stenus trivialis* Kraatz | 5 | 17 | 1 | 3 | 26 | 2 | 6 | | | 8 |
| *Tachyporus hypnorum* (Fabricius) | 1 | | | | 1 | | 12 | | | 12 |
| *Tachyporus nitidulus* (Fabricius) | 1 | 4 | | | 5 | 3 | 3 | | | 6 |
| *Tachyporus* sp.1 | | | | 2 | 2 | | | | | |
| *Tachyporus* sp.2 | 1 | | | | 1 | | | | | |

**Supplementary material Table S3.** List of rove beetle species sampled by pitfall traps. GM = green manure, RC = roller crimper. (continued)

| | Cauliflower | | | | | Tomato | | | | |
|---|---|---|---|---|---|---|---|---|---|---|
| | 2015 | | 2016 | | | 2016 | | 2017 | | |
| **Species** | **RC** | **GM** | **RC** | **GM** | **Total** | **RC** | **GM** | **RC** | **GM** | **Total** |
| *Tasgius falcifer* (Nordmann) | 3 | 6 | 9 | 10 | 28 | | | 2 | 1 | 3 |
| *Tasgius globulifer* (Geoffroy) | | 10 | | | 10 | | | | | |
| *Tasgius pedator* (Gravenhorst) | 3 | 1 | 23 | 15 | 42 | | 1 | | | 1 |
| *Tasgius winkleri* (Bernhauer) | 8 | 3 | 13 | 21 | 45 | | | | | |
| *Xantholinus appenninicola* Steel | | | 1 | 5 | 6 | | | | | |
| **Total** | **188** | **546** | **278** | **465** | **1477** | **47** | **120** | **11** | **20** | **198** |

**Supplementary material Table S4.** List of spider families sampled by pitfall traps. GM = green manure, RC = roller crimper.

| | Cauliflower | | | | | Tomato | | | | |
|---|---|---|---|---|---|---|---|---|---|---|
| | 2015 | | 2016 | | | 2016 | | 2017 | | |
| **Family** | **RC** | **GM** | **RC** | **GM** | **Total** | **RC** | **GM** | **RC** | **GM** | **Total** |
| Agelenidae | | | | | | 1 | 1 | | | 2 |
| Cheiracanthiidae | 1 | | | | 1 | | | 2 | | 2 |
| Cybaeidae | | 1 | | | 1 | | | | | |
| Dysderidae | 12 | 9 | 30 | 30 | 81 | 5 | 7 | 5 | 9 | 26 |
| Gnaphosidae | 79 | 42 | 27 | 27 | 175 | 27 | 46 | 78 | 40 | 191 |
| Linyphiidae | 10 | 17 | 19 | 25 | 71 | 104 | 18 | 45 | 23 | 190 |
| Liocranidae | 1 | 2 | 1 | | 4 | | 5 | 4 | 1 | 10 |
| Lycosidae | 193 | 282 | 321 | 250 | 1046 | 442 | 1119 | 311 | 659 | 2531 |
| Mimetidae | | | | | | 1 | 1 | | | 2 |
| Nemesidae | | | 1 | 4 | 5 | | | 1 | 1 | 2 |
| Oecibiidae | | | | | | | | | 1 | 1 |
| Philodromidae | | | | | | | | 2 | 1 | 3 |
| Pholcidae | | | | | | | | 1 | | 1 |
| Phrurolithidae | | 3 | 1 | 5 | 9 | 1 | 2 | 1 | 1 | 5 |
| Salticidae | 3 | 5 | 1 | 4 | 13 | 4 | 1 | 4 | 4 | 13 |
| Tetragnathidae | | | | | | 1 | | | | 1 |
| Theridiidae | 1 | | | 1 | 2 | | | 10 | 4 | 14 |
| Thomisidae | 17 | 10 | | | 27 | | | | | |
| Titanoecidae | | | 5 | 3 | 8 | 5 | 4 | 3 | 3 | 15 |
| Trachelidae | 1 | 2 | | 1 | 4 | | | | 1 | 1 |
| Zodariidae | 1 | | 1 | | 2 | | | 5 | 4 | 9 |
| **Total** | **319** | **373** | **407** | **350** | **1449** | **591** | **1204** | **472** | **752** | **3019** |
